# Access to Medicines, Access to Markets

**DOI:** 10.3389/fsoc.2020.00058

**Published:** 2020-08-14

**Authors:** Sergio Sismondo

**Affiliations:** Department of Philosophy, Queen's University, Kingston, ON, Canada

**Keywords:** access to medicines, access to markets, pharmaceutical industry, marketing, drugs

## Abstract

This article explores some uses by the pharmaceutical industry of language from the “access to medicines” movement in global health, sometimes for goals almost completely opposite to those of the movement. Important in the context of extremely expensive treatments, the industry draws on the idealistic discourse around access to medicines to create a very specific continuity between the needs of the Global South and its own marketing needs. By focusing on “access,” the industry can promote the opening up of markets in relatively wealthy countries with important public or highly regulated payers.

## An Opportunity and a Problem For Pharmaceutical Companies: Sky-High Prices

In May of 2019, Novartis won FDA approval for Zolgensma, a gene therapy for spinal muscular atrophy, a group of rare, degenerative and often-fatal conditions. The agency approved the one-time treatment for patients under 2 years of age who had a particular mutation in the SMN1 (survival motor neuron) gene (Bosely, 2019). Zolgensma attracted some attention for being one of very few approved gene therapies, one of only two then available in the US. But it attracted considerably more attention as a result of its price: slightly more than US $2 million for a treatment, setting a new record for a drug!

That might look like a record that a company would not want to hold, but Novartis didn't appear concerned. The record had changed hands many times over the previous decade, a decade that started with sticker shock at treatment prices of a mere US $100,000, amounts that shortly became commonplace. For example, the cancer drug Provenge was priced at US $93,000 in 2011, a price that led to low sales because physicians and hospitals were concerned about being reimbursed (Fuerstein, 2011). In the pharmaceutical industry, the 1990s and 2000s were marked as the decades of blockbuster drugs—earning more than US $1 billion in a year—for widespread use. The 2010s, by contrast, were the (first?) decade of extravagantly priced drugs for narrow use.

Clearly, sky-high prices for drugs are attractive for pharmaceutical companies. Equally clearly, they present the problem that customers, including different kinds of insurers, may not be willing to pay for those drugs. It is not a foregone conclusion that anybody will pay $100,000 for a treatment, let alone $2 million. Over the past decade, as they have introduced higher and higher prices, companies have had to work on solving this problem.

In the case of Zolgensma, the record price had been thought through. As a one-time treatment, the US $2 million is about the same as 5 years of successful treatment with a competing drug, Spinraza, made by Biogen. Given the precedent that Biogen had established, Novartis's pricing was somewhat less bold than it looked. Moreover, at the broader level, some of the rhetorical work to address the problem had already been done. Most US private insurance plans and Medicaid will cover Zolgensma's cost for at least a subset of the small number of spinal muscular atrophy patients. For the US insured patients, the question of access to this medicine has already been at least partly answered, as has the question of access to this market for Novartis.

In addition, Novartis announced plans to donate, through a lottery, 100 doses of Zolgensma per year to children outside the US. The geographical choice is interesting, because it recognizes that Zolgensma's price puts it out of the reach of almost everybody. This lottery program accomplishes a number of things at once. It allows Novartis to claim that it is promoting access to medicines, while hiding from Zolgensma's prize market, insured patients in the US, the lower prices Novartis might be forced to charge elsewhere (see Ecks, 2015 for analysis of an analogous case). And the lottery will likely generate some valuable advertising for the treatment in Western Europe and elsewhere—and some mobilization of patient groups to get it covered by public and other insurance plans.

I should note that there was a potential glitch in the approval process. Although results from the clinical trial of 68 children were positive, it was found that the company originating the drug, AveXis, had manipulated or falsified data from animal testing. Neither Novartis nor AveXis revealed this fact until 1 month after FDA approval (FDA, 2019). Their failure to report the manipulated data was potentially consequential. The FDA (FDA, 2019) wrote in a press release another month later: “The agency will use its full authorities to take action, if appropriate, which may include civil or criminal penalties.” Two AveXis employees were fired, but it is unclear exactly how the FDA has acted. The animal data concerns were serious enough that the FDA ordered a halt to a later study, though they didn't affect approval. An AveXis spokesperson said that the data problems had been fully explained to the FDA and didn't affect “the medicine itself” (Bosely, 2019).

With approval, some cases covered, and a lottery program on the way, Novartis could consider the most immediate issues of access to be dealt with—and indeed, almost 100 infants, earning the company revenue of US$160 million, were treated in the financial quarter immediately following availability in the US (Dunn, 2019).

The lottery for Zolgensma is only one focused face of Novartis's efforts at access. The company has developed programs for a number of its other expensive drugs (e.g., Ecks, 2015). Most prominent at the moment is probably its “Novartis Access” program, which offers 15 drugs for non-communicable diseases at a rate of $1 per month, in a number of countries in sub-Saharan Africa, Central America, Southeast Asia and Central and Eastern Europe (Novartis, 2020). Many of those drugs are either on or are competing with drugs on the World Health Organization's (WHO) “Model list of essential medicines” (WHO, 2019). Novartis Access is the flagship of the company's “Social Business” unit, and can boast of hundreds of thousands of treatments per year. The evidence for effects of Novartis Access on the ground is mixed (Rockers et al., 2019), though there is no doubt that it is a public relations success.

Novartis's efforts to get approval for Zolgensma, its promoting and pricing of the treatment in the US, its program of donating a small number of treatments outside the US, and its Novartis Access program, can be seen as different approaches to access. While these approaches are genuinely different, they also occupy the same terrain. The pharmaceutical industry has adapted to the discourse of access to medicines, and has adopted some of the key terms of that discourse to its own ends.

## The Access to Medicines Movement

Greene (2011) insightfully asks, how did a discourse around “essential drugs”—later “essential medicines”—arise and become dominant? Why did *drugs* become essential? Greene's answer has many parts, and I will skip to a central feature of it. Although there were antecedents, the WHO's first publication of a “model list of essential drugs” (in WHO, 1977, p. 20–33) is the key event that shaped debates around what was essential and what not. This particular list was born in the context of a conflict between low-income countries and multinational pharmaceutical companies over the cost of drugs, and followed a call for a shift in the production of drugs to members of the Non-Aligned Movement (Greene, 2011, p. 17). The objects—drugs—at the center of the conflict thus became elevated in importance, to the point of being made “essential.” Clearly, the problem of access to medicines is implicit in the publication of a list of essential medicines, but it took two decades to come to the fore, and to be generalized.

**Figure 1:**
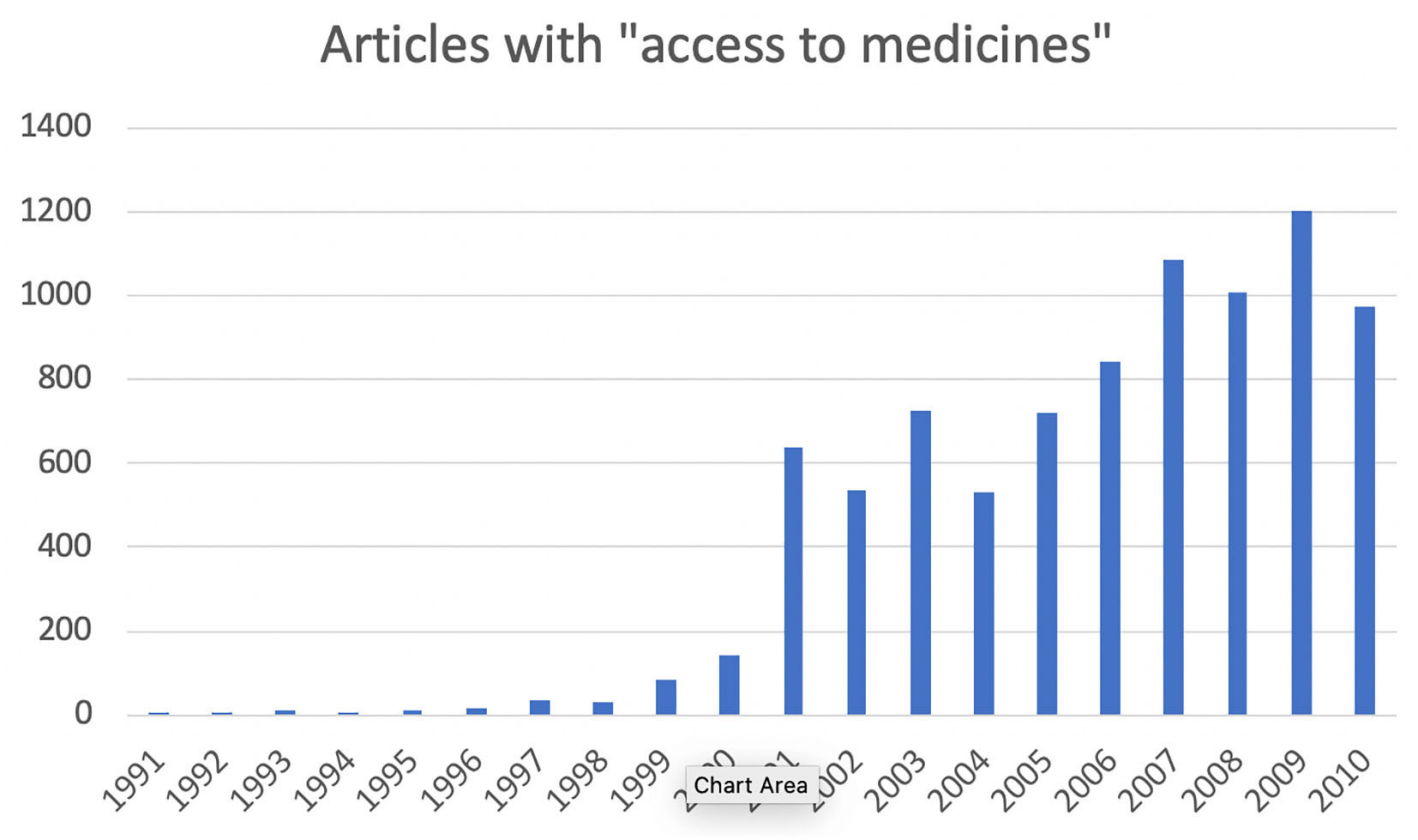
Articles mentioning “access to medicines,” 1991–2010 (*Factiva* database, search performed 19 June 2020).

We can see dramatic growth in use of the phrase “access to medicines,” beginning in the second half of the 1990s (Figure 1). Moreover, the applications of the phrase become narrower at about that time, more focused on the problem of drugs for treating life-threatening conditions that are unaffordable, especially in developing countries. In terms of more academic interest, Google Scholar and Google Ngram show essentially the same pattern of results.

The growth in the use of the phrase reflects activism and debates around the essential drugs concept, but in the form of an access to Medicines movement sparked by the TRIPS (Trade-Related Aspects of Intellectual Property Rights) Agreement. The story of TRIPS, including the central role of a number of large pharmaceutical companies in the design of TRIPS, has been well told many times (e.g., Drahos, 1995; Sell, 2001; Matthews, 2003). In the early 1980s, a group of companies successfully put intellectual property at the top of the US trade agenda and helped to design not only international agreements, but also a very successful strategy for convincing other countries to sign onto them. Prominent in that group of companies were representatives of the pharmaceutical industry: the Advisory Committee for Trade Negotiations, formed in 1981, was chaired by the CEO of Pfizer, Ed Pratt, and its 1986 successor, the Intellectual Property Committee, consisted of 13 large companies including Pfizer, Bristol-Myers, Johnson & Johnson, and Merck, making pharmaceuticals the largest of the industry sectors represented on the committee; the chemical, computer, automotive, aerospace, and communication industries also were represented. These committees designed a strategy that combined the building of coalitions with non-US companies, forceful bilateral pressure to link intellectual property and other trade, and finally multilateral agreements built on those bilateral pressures. The result was the 1995 TRIPS Agreement, which has formed the basis for new intellectual property laws in countries around the world. Effectively, TRIPS extended a version of a new and strengthened US intellectual property law to many parts of the globe.

In the wake of TRIPS, a campaign for access initially involved the Consumer Project on Technology, Health Action International, and Médecins Sans Frontières though expanding to include a number of other organizations (Sell, 2001). A notable event was the Conference on Increasing Access to Essential Drugs in a Globalized Economy, taking place in 1999 in Amsterdam; the conference issued a statement that connected TRIPS and the immense challenges of global health, and calling on the World Trade Organization to establish, at its upcoming meeting in Seattle, a standing working group to address the issue (WHO, 1999). TRIPS and “access to medicines” were so strongly linked that in the early 2000s almost all of the most prominent discussions of access also referred to TRIPS or to patent protection. The most immediate concerns that linked the two were about antiretroviral treatments, given the HIV/AIDS crises in sub-Saharan Africa, Southeast Asia and Brazil. The concerns were realized when, for example, the US government used all of its legal power to try to intimidate South Africa into repealing its legislation allowing compulsory licensing of generic HIV/AIDS drugs (Sell, 2001, p. 501ff). When the US government withdrew from the confrontation, the pharmaceutical industry attempted legal action on its own, but also backed away in the face of domestic protests. Individual companies relented, and adjusted their pricing of the drugs in different countries. “Access to medicines” was a public relations mess for the pharmaceutical industry in the early years of this century (e.g., 't Hoen, 2003). The industry recognized this, and immediately engaged in damage control, trying to reframe the problem as arising because of a regrettable combination of their high research and development costs and poverty in the Global South (e.g., Cochrane, 2000). Within a few years, though, companies were systematically setting up access programs to try to clean up their image messes (Greene, 2011, p. 25–26).

Setting the context for the discourse on access to medicines, the World Health Organization's current highest-level webpage on the subject begins: “Nearly 2 billion people have no access to basic medicines, causing a cascade of preventable misery and suffering” (WHO, 2020). The statement continues:

Good health is impossible without access to pharmaceutical products. Universal health coverage depends on the availability of quality-assured affordable health technologies in sufficient quantities. … Efforts to improve access to medicines are driven by a compelling ethical imperative. People should not be denied access to life-saving or health-promoting interventions for unfair reasons, including those with economic or social causes (WHO, 2020).

As this suggests, the conflict and the issue have not gone away. And it is also possible that, like lists of “essential medicines,” conflicts about “access to medicines” make drugs in general all the more prominent in discussions about global health. But unlike “essential medicines” or possible alternatives such as “affordable medicines,” the focus is on *access*, a concept with some flexibility.

## Access Matters to Pharmaceutical Companies

Clearly, the pharmaceutical industry, which makes few of its products affordable in poor countries, is one of the central problems, perhaps *the* central problem, identified by the access to medicines movement. As a result of the large public relations challenges, pharmaceutical companies have developed a number of programs to improve public relations by making some products available to some populations at relatively low costs. The Novartis Access program mentioned above is one, but every major company has multiple such programs, blunting some of the effect of the access to medicines movement.

However, the industry also has made positive use of some of the language of access to medicines: not to laud its access programs or to advocate for better healthcare in poor countries, but as part of efforts to convince payers in wealthy countries to cover expensive drugs, to cover more patients for their drugs, and to cover them more quickly. The industry has picked up the ideals and the discourse around access, and has applied those to situations in which it can most profit.

For example, in a report on “Access to new medicines in public drug plans,” the industry lobby group Innovative Medicines Canada goes so far as to lead by citing its former opponent the WHO:

According to the World Health Organization (WHO), access to medicines and vaccines is a key component to a quality health system. There is no doubt that innovation in medicines and vaccines has made a significant contribution to improving health outcomes in Canada and around the world. It is therefore important for Canadians to know the state of access to new medicines in their country, relative to comparable countries. The goal of this study is to measure access against international benchmarks in order to drive improvements to access here at home (Innovative Medicines Canada, 2016, p. 1).

Here, selectively adopting the language of access to medicines allows this industry association to make the case that Canadians could be better served. Only 37% of new medicines were reimbursed across all provinces (public drug plans are run by individual provinces, not the Federal government), and most involved some “reimbursement conditions.” Even excluding smaller provinces, only 59% of new cancer medicines and only 23% of biologics were covered. There is an average wait time of 449 days between drug approval and the beginning of reimbursement. On almost every one of the report's metrics, Canada places poorly among comparator countries, and readers can only conclude that there is a serious problem of access to medicines in the country.

The Association of the British Pharmaceutical Industry's website also draws attention to access: “For every 100 patients that get a new medicine in its 1st year of launch in other parts of the EU—including France and Germany—just 21 patients in the UK get access. There is also significant variation across the UK when it comes to accessing different types of medicines” (ABPI, 2020). The German Medicines Manufacturers' Association (BAH) has a report on “Sustainable access to medicines in Europe” (BAH, 2017). Unlike the Canadian and UK reports, this one generously focuses on patient access to medicines outside Germany, arguing for increased harmonization of regulations and new trade deals—for example with the UK following Brexit.

Important in the background here is the rise of health technology assessment (HTA), used in many jurisdictions to rationalize (especially) the public provision of healthcare services and treatments. Among other things, HTA does cost-benefit analyses of drug treatments, and in some places these analyses translate almost directly into decisions about which drugs are funded systematically. Across Europe, for example, there are national HTA agencies. The kinds of evidence assessed by these agencies are broadly similar, and their roles are similar, though there are significant differences in the agencies' analyses and how they are applied (Angelis et al., 2017).

Nonetheless, “[i]n recent years, access to essential medicines has become an issue even in the wealthiest parts of Europe. In particular, the proliferation of high-priced medicines has pushed the issue of access to new medicines high on the policy agenda of all European countries, including in high-income economies” (Vogler et al., 2017). As a result, though HTA establishes the baselines for healthcare systems' funding of drugs, there are multiple and various alternative access schemes (Löblová et al., 2019). Often as a result of lobbying, unapproved drugs may be funded for “compassionate use,” or “off-label” or more particular schemes, and approved drugs may be made available through special funds or on a putatively trial basis awaiting further evidence of effectiveness. When they don't manage to land their products on public formularies, it is in pharmaceutical companies' interests to encourage or lobby for some kind of alternative access scheme.

Patient advocacy organizations (PAOs) have often been excellent vehicles for promoting specific ideals of access in wealthy countries (O'Donovan, 2007), and over the past few decades pharmaceutical companies have become increasingly aware of the value of PAOs as such vehicles (Sismondo, 2018). In forming alliances, PAOs are likely to cede some control over the uses of their efforts and the public articulation of their interests, though they may still get meaning and value from their actions (see, e.g., Klawiter, 2008). Of relevance here, specific groups of patients may have strong interests in promoting high-priced drugs, when, for example, companies provide the drugs to those groups for free or at a greatly reduced price—through a special access program (e.g., Ecks, 2015). Some single-disease PAOs have been important allies in companies' efforts to secure quick regulatory approval for specific new drugs, even when the data is equivocal.

Thus companies generously fund PAOs, hoping to align interests. For a 2018 news story on payments to PAOs—now a well-reported phenomenon—a spokeswoman for the company said that “Bristol-Myers Squibb is focused on supporting a health care environment that rewards innovation and ensures access to medicines for patients. The company supports patient organizations with this shared objective” (Kopp et al., 2018). Today, patient voices are represented in almost every controversial drug approval process, and they are speaking the language of access.

When the UK government commissioned an “Accelerated Access Review,” the reviewers heard from a large number of patients and PAOs, and, at least as represented in the review, they were overwhelmingly in favor of streamlined approvals for some classes of drugs (Accelerated Access Review, 2016, see also Muscular Dystrophy UK, 2015). The resulting Accelerated Access Review program was indeed tasked with bringing innovative treatments to patients more quickly; it was initially headed by the former CEO of GlaxoSmithKline, Andrew Witty, then 6 months out of his previous position (Jefferson, 2017). It appears that today's PAOs formed around one or a cluster of debilitating or life-threatening diseases are very likely to advocate in favor of early approval of new drugs, or even compassionate use for substances that have not yet been approved—on the latter, pharmaceutical companies can even reasonably claim to be more conservative than patients and PAOs (e.g., Pharmaphorum, 2014).

Of course, some of the PAOs involved are even more closely connected with the pharmaceutical industry than they reveal. I provide a lengthy example. In 2017, the prominent health newsletter *STAT News* published an op-ed by Dr. Robert Yapundich (2017), a neurologist, who argued that sales reps should be allowed to discuss “off-label” uses of drugs—uses for which the drugs aren't approved. This, he said, drawing on anecdotes about patients, would allow him to better help his patients. Yapundich's bio mentioned that he was a member of a US group called the Alliance for Patient Access. Another newsletter, HealthNewsReview (2017a), quickly noted that Yapundich had accepted a considerable amount—more than $300,000, as it turned out—from the drug industry, and hadn't noted the conflict of interest. Embarrassed by these and other revelations, *STAT News* withdrew the article.

Although the name, Alliance for Patient Access, suggests a PAO, it is officially an organization of physicians. The physicians who sit on the organization's Executive include some of the industry's most highly paid key opinion leaders, including Dr. Srinivas Nalamach, who received $800,000 from drug companies between 2013 and 2015, in connection with the promotion of opioids and drugs to treat the side effects of opioids (HealthNewsReview, 2017b).

In addition to not reporting his conflicts of interest, Yapundich had neglected to mention that the article was drafted for him by a public relations firm working for the Alliance (HealthNewsReview, 2017c). Yapundich stood by the article, though he acknowledged that the ghostwriters had either fabricated or made mistakes about some details of the anecdotes. It turns out the Alliance for Patient Access is actually operated by the public relations firm that commissioned the ghostwritten op-ed, and that the Alliance is supported primarily by membership dues paid by pharmaceutical companies and trade associations. So, what is superficially a patient organization is officially a physician organization and is effectively a pharmaceutical industry organization—or at least a creature of the industry. It is unsurprising, then, that the Alliance for Patient Access opposes limits on drug costs, even though high costs clearly affect patients' access to drugs. For example, in the midst of a public outcry over steep drug price hikes, the Alliance for Patient Access wrote a blog post on the need for a “comprehensive dialogue,” especially focused on how insurers should cover full costs of drugs (Alliance for Patient Access, 2015). When discussions at a United Nations panel on access to medications turned to exorbitant costs as a result of patents, the Alliance wrote a blog post on how patents make access possible (Alliance for Patient Access, 2016).

As we've already seen, strong intellectual property laws tend to create monopolies that allow for very high prices. Nonetheless, there is no shortage of PAOs willing to advocate in favor of patent protections for pharmaceuticals, in the name of increased innovation. In response to discussions on a United Nations panel, which pointed fingers at drug patents as key culprits in keeping needed drugs expensive and out of the hands of patients, fifty PAOs wrote to then-Secretary of State John Kerry, to support the US government's strong defense of the patent system. Some of those organizations might have been acting as a result of hopes for magic bullets, and some might have been acting purely as creatures of the pharmaceutical industry: the Global Alliance for Patient Access, a spin-off project of the US Alliance for Patient Access, was one of the signatories (Global Colon Cancer Association, 2016).

The access to medicines movement, including the organizations that began it, continues in its efforts to contribute to global health by advocating and acting for the affordability of important drugs, especially in the Global South, and to oppose intellectual property regimes that stand in the way of those efforts. In this case, activists in the movement have been able to remain independent of the pharmaceutical industry and its actions.

But with newer groups like the US and Global Alliances for Patient Access, the motivations behind the original access to medicines movement have been turned on their heads. The pharmaceutical industry is arguing, using supposed patient advocacy organizations as its mouthpieces, in favor of unfettered access to consumers in wealthy countries, as well as higher prices and stronger intellectual property regimes. The companies are busy rhetorically establishing a very specific continuity between issues in the Global South and the North, a continuity that can be made precisely because of the extremely high prices that the companies want to charge.

## Access to Markets

But nothing in the adoption of the access discourse should be surprising. From the point of view of the industry, patients' access to medicines is essentially the same as companies' access to markets, though the value of drawing attention to them is very different.

The American Marketing Association (2017) has a broad concept of marketing, defining it as “the activity, set of institutions, and processes for creating, communicating, delivering, and exchanging offerings that have value for customers, clients, partners, and society at large.” In the “marketing era” (Applbaum, 2004) captured by this expansive definition, products and services don't simply arrive at a marketplace to be sold. In the ideal case, every step in the trajectory of manufacture, advertisement, transportation, sale, delivery and consumption will have been shaped by every other step. In the context of the pharmaceutical industry, the American Marketing Association's definition would include anything that pharmaceutical companies do to get their products into consumers' bodies. As a result, not only is patients' access to medicines conceptually linked with companies' access to markets, but the work that companies do to increase access to medicines is part of their marketing of those products.

In a short article for pharmaceutical marketers in Europe and the UK, insider Colin Wright provides a view of market access that firmly links it to access to medicines. He starts with a definition: “Market access is the process to ensure that all appropriate patients who would benefit, get rapid and maintained access to the brand, at the right price” (Wright, 2012, emphasis removed).

Similarly, a survey of UK industry employees working on market access produced a very similar definition of the term: “Ensuring patients receive appropriate treatment at the right time and right price, working with the local/regional NHS and their processes based on value” (Bradley, 2017). Marketer Craig Bradley writes: “Essentially, there is a need for interested stakeholders to work together to develop a system that is fit for purpose in recognizing innovation and allowing patient access to new treatments that can demonstrate value. The main issue is making sure that patients are able to access innovative new treatments.” As he notes, this is operationalized by the UK Pharmaceutical Marketing Society as: “Principally market access involves preparing a positive environment which supports uptake of your product and demonstrating the ‘value' of your product to the range of customers who influence uptake. Strategically, market access is about packaging data in the right way, for the right customer at the right time” (PM Society 2020). Access to medicines and access to markets are linked, or even fused.

On the expansive view of marketing above, regulatory approval itself—whether accelerated or not—is a crucial element of access. Approval represents the first possible date for market access, though in most cases there is a delay. In his article on market access, Wright (2012) provides a chart showing the average number of days between “marketing authorization” and “patient access” for a number of European countries—ranging from zero days in the case of the UK and Germany to 392 days in the case of Belgium. The chart is quite similar in content and form to ones in the Innovation Medicines Canada report on “access to medicines.”

After regulatory approval, then, pharmaceutical companies have to work with payers, respecting their processes and identifying the value that a new drug can offer. In launching a drug, companies are “preparing the brand for the market, preparing the company for the brand.” But this is a matter of “value communication,” and in particular is a matter of identifying “improved health outcomes: outcomes that reflect the correct endpoints in eyes of payers.” And thus market access needs to focus on patients. Wright emphasizes points that would be at home in many treatments of access to medicines: “Benefit must be expressed relative to Standard of Care…. Preferably, it would be expressed in real-life settings to show how the new medicine performs in more naturalistic environments, which reflect the value that will be delivered in real life.” And, there is no one-size-fits-all solution, as the “market access process must link the requirements at global level, which guide the clinical development process, to the needs at local country level” (Wright, 2012, emphasis removed; also Proctor and Silvey, 2017). Again, access to medicines and access to markets are linked.

And thus, Joseph Jimenez, CEO of Novartis can say: “Innovation doesn't just mean developing new drugs. So innovation also, in our minds, includes new business models that can improve access to medicines to people around the world” (Novartis, 2015). This is a way of speaking that makes perfect sense in the context of deep concerns with access to medicines/access to markets.

## Author Contributions

SS did the research, planned, and wrote the manuscript.

## Conflict of Interest

The author declares that the research was conducted in the absence of any commercial or financial relationships that could be construed as a potential conflict of interest.
